# Entomo-virological surveillance of *Flavivirus* in mosquitoes in Yucatan State, Mexico

**DOI:** 10.1590/S1678-9946202466056

**Published:** 2024-09-23

**Authors:** Diana Guadalupe Argaez-Sierra, Carlos Marcial Baak-Baak, Julian E. Garcia-Rejon, Rosa Carmina Cetina-Trejo, Julio C. Tzuc-Dzul, Karla Y. Acosta-Viana, José I. Chan-Perez, Nohemi Cigarroa-Toledo

**Affiliations:** 1 Universidad Autónoma de Yucatán, Centro de Investigaciones Regionales “Dr. Hideyo Noguchi”, Laboratorio de Arbovirología, Mérida, Yucatán, Mexico; 2 Universidad Autónoma de Yucatán, Centro de Investigaciones Regionales “Dr. Hideyo Noguchi”, Laboratorio de Biología Celular, Mérida, Yucatán, Mexico

**Keywords:** Aedes aegypti, Zika virus, Phlebotomus-associated flavivirus, Insect-specific flaviviruses

## Abstract

The genus *Flavivirus* (Family: *Flaviviridae*) comprises arboviruses with the capacity to infect humans and animals. It also integrates insect-specific viruses. This study aimed to identify *Flavivirus* in mosquitoes captured in 17 municipalities in Yucatan State, Mexico. The mosquitoes were caught in households from November 2021 to May 2022. A total of 4,321 adult mosquitoes from five species were caught. The most abundant were *Culex quinquefasciatus* (n = 3,563) and *Aedes aegypti* (n = 734). For molecular investigations, 600 female mosquitoes were split into groups of 10, mostly for species and site location. Reverse transcriptase polymerase chain reaction (RT-PCR) amplified a region of the NS5 gene to find the *Flavivirus* ribonucleic acids (RNA). A total of 24 pools that were positive for *Flavivirus* were detected in *Ae. aegypti* specimens and subsequently subjected to sequencing using the Sanger method. A total of 12 sequences matched the established quality criteria and were subsequently employed for sequence homology analysis. We found that one sequence corresponded to the Zika virus (ZIKV), and 11 sequences had sequence similarity with Phlebotomus-associated flavivirus (PAFV), an insect-specific virus (ISF). In conclusion, we found ZIKV in the Merida municipality, Yucatan State, which suggests that the virus is silently circulating. Phlebotomus-associated flavivirus is distributed in five municipalities in Yucatan State, Mexico. Future studies could focus on isolating this virus and studying its biological role within *Ae. aegypti*.

## INTRODUCTION

Arboviruses (a term derived from arthropod-borne viruses) are a diverse group of viruses that are potentially infectious to humans and animals when they are transmitted by arthropod vectors (mosquitoes, ticks, sandflies, or biting midges)^1^. More than 600 arboviruses have been discovered; these are integrated into six families. Most arboviruses that cause human diseases belong to the families *Flaviviridae, Togaviridae*, and *Peribunyaviridae* ^2^. The studies on *Flavivirus* in arthropods have concentrated on those that are associated with diseases in vertebrates. This emphasis stems partly from the fact that hosts who are vertebrates typically show more visible signs of infection^1^. The genus *Flavivirus* encompasses 53 species of viruses and contains those transmitted between hematophagous arthropods and vertebrate hosts, viruses with no known vector (NKV), and insect-specific flavivirus (ISF)^3-5^. Flaviviruses that infect vertebrates can be categorized into two groups: mosquito-borne flaviviruses (MBFVs), which include dengue virus, Zika virus, and West Nile virus^5^; and tick-borne flaviviruses (TBFVs), which include tick-borne encephalitis virus and Powassan virus^6^. The viruses with no known vector have been isolated in nature from bats, rodents, and occasionally humans, but never from wild-caught or laboratory-inoculated arthropods or arthropod cell cultures. Viruses with no known vector can be categorized into two groups: flaviviruses linked with bats and flaviviruses linked with rodents^3,7^. Infections from the group encompassing insect-specific-flaviviruses show no visible signs of infection in the arthropods they infect. It also does not infect animals or vertebrate cell cultures; however, viruses such as the Aedes flavivirus (AEFV) can produce a mild cytopathic effect in mosquito C6/36 cell^4,5^.

In nature, mosquitoes are the primary source of ISFs. However, they have also been identified in bees, cockroaches, chironomids, and sandflies, suggesting the potential for infection in other arthropods^5^. A number of these viruses have been unintentionally identified by the use of generic primer sets, specifically those that are intended to detect a segment of the non-structural protein NS5 found in flaviviruses^8-11^. Insect-specific flaviviruses can be classified into two categories: classical ISFs (cISFs) and dual-host affiliated ISFs (dISFs). The cISFs were the first to be discovered as a clade different from the others^4^. The cell-fusing agent virus (CFAV) represents the group^12^. An interesting fact is that the dISFs are phylogenetically related to the mosquito-borne flaviviruses, despite their inability to replicate in vertebrate cells^4,5^.

In Mexico, there is active circulation of the dengue virus, with occasional occurrences of the Zika virus^13^. During monitoring of infectious flaviviruses in vertebrates and mosquitoes, the ISFs Culex flavivirus (CxFV) and T’Ho virus have been frequently detected in *Cx. quinquefasciatus* ^14-17^. In field or experimental studies, it has been shown that ISFs show the capacity to modulate or enhance the co-infection, replication, and transmission of flavivirus^18-20^. In an endemic focus on West Nile virus (WNV) transmission in Chicago city, United States, it was discovered that 40% of *Culex* mosquitoes who were WNV-positive were also CxFV-positive, demonstrating that both viruses can co-infect mosquitoes in nature^18^. In a controlled laboratory study, it was demonstrated that the Nhumirim virus (NHUV) can inhibit the replication of the West Nile virus in *Cx. quinquefasciatus* mosquitoes when they are infected with both NHUV and WNV^19^. Similarly, it was observed that NHUVs suppress the viral replication of Zika virus up to 100,000 times and dengue virus-2 up to 10,000 times in *Aedes albopictus* cells^20^.

Prior to the coronavirus pandemic, Mexico showed 501,600 recorded cases of dengue from 2007 to 2020, and the virus was linked to 1,230 deaths^13^. However, the incidence of diseases spread by vectors, such as dengue, significantly decreased during the peak of SARS-Cov-2 transmission^21^. Our hypothesis was that the flaviviruses were circulating despite the underreporting of cases. Therefore, this study aimed to conduct entomo-virological surveillance of flaviviruses in mosquitoes from households in Yucatan State, Mexico, after the critical period of the pandemic.

## MATERIALS AND METHODS

### Study area

**Figure 1:**
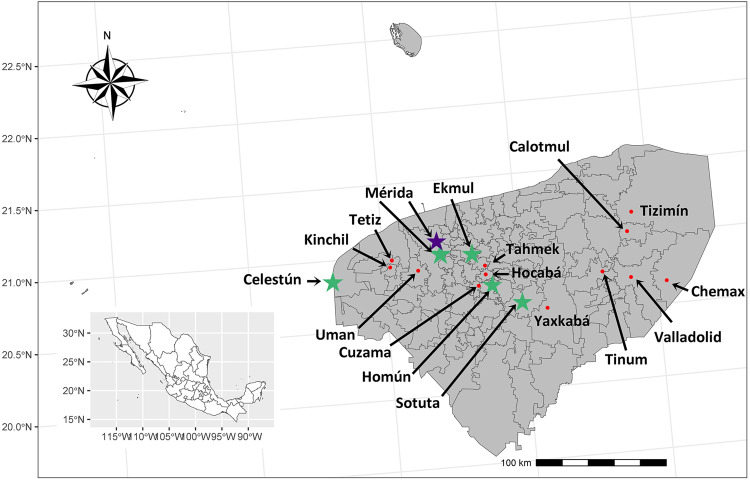
Municipalities of Yucatan State, Mexico, that were visited to capture mosquitoes. The green star represents the municipality with mosquitoes positive for Phlebotomus-associated flavivirus, the purple star represents the pool positive for Zika, and the red circles are the municipalities with mosquitoes negative for *Flavivirus*.

The entomo-virological study was conducted in 17 municipalities located in Yucatan State (Figure 1). Yucatan State is located on the Yucatan Peninsula in Mexico and shares borders with Quintana Roo State and Campeche State (20°50’0” N, 89°0’0” W). The rainy season (May to October) records an average rainfall of 1,000 millimeters and an average temperature of 27.58 °C. The dry season extends from November to April, with an average precipitation of 300 millimeters and an average temperature of 25.18 °C^22^.

### Design and capture of adult mosquitoes

A cross-sectional study was conducted. In each location, seven to 22 households were visited. The houses were chosen based on their convenience. Sampling was carried out in areas where flaviviruses circulation and mosquito abundance were previously reported^22-25^. Mosquito capture was performed from November 2021 to May 2022 with the aid of a Propack-type backpack-mounted aspirator (John W. Hock Company, Gainesville, US). Every room in each house was subjected to mosquito trapping, with a focus on dark locations such as behind furniture, within closets, and on curtains. Backyards were also included, particularly the shady parts (tool sheds and pet shelters). Adult mosquitoes were placed in plastic containers labeled with an identification number, collection date, and site sample. Mosquitoes caught were transported alive to the Arbovirologia laboratory at the Universidad Autonoma de Yucatan and identified up to species using stereomicroscopes and taxonomic keys^26^.

### RNA extraction and molecular assays

To perform flaviviruses detection tests, the mosquito species with the highest abundance of females were selected, including the *Ae. aegypti* (n = 260), *Cx. quinquefasciatus* (n = 328), and *Ae. albopictus* (n = 12). A female mosquito was put into each Eppendorf microtube, and then 50 µL of the DNA/RNA Shield^TM^preserver (Zymo Research) was added. Then, the samples were stored at −80 °C until they were used in molecular analyses. Afterwards, each mosquito was mechanically homogenized with sterile pestles, and 50 µL of Eagle’s minimal essential medium (Invitrogen, Carlsbad, CA, USA). Homogenates were centrifuged at 10,000 × g for 7 min. In total, 15 µL of each individual supernatant was collected to create pools of 10 mosquitoes (150 µL). Female mosquitos were pooled according to species and capture site.

Total RNA was extracted from each pool (aliquot 120 µL) using a QIAamp Viral RNAkit (Qiagen, catalog Nº 52906. Valencia, CA, US) following the manufacturer’s protocols. Complementary DNA (cDNA) was synthesized with 650 ng of total ARN samples using a commercial kit ADNc RevertAid H Minus First Strand (Thermo Scientific). The semi-nested pan-flavivirus PCR protocol described by Scaramozzino *et al*.^11^ was followed to detect *flavivirus* RNA. The primers cFD2 (5’-GTGTCCCAGCCGGCGGTGTCATCAGC-3’), MAMD (5’-AACATGATGGGRAARAGRGARAA-3’), and FS778 (5’-AARGGHAGYMCDGCHATHTGGT-3’) were used to amplify a ~250 nucleotide region of the NS5 gene^11^. For a 25-µL PCR reaction, 3 µL of ADNc, 2 µL of MgCl_2_ at a concentration of 25 mM, 2.5 µL of 5X reaction buffer, 0.2 µL of dNTPs, 0.5 µL of dNTPs, 1.5 U of Taq polymerase (Invitrogen), and 0.5 µL of each primer at a concentration of 10 µM were added, with the rest of the volume being filled with nuclease-free water. Negative (molecular biology-grade water) and positive controls (DENV-2) were included in each PCR. Thermal cycling conditions consisted of denaturation at 94 °C for 5 min, hybridization at 35 cycles at 94 °C for 1 min, 54 °C for 1 min, and 72 °C for 1 min, followed by a final elongation at 72 °C for 5 min. Amplicons were visualized on 1.5% agarose gels with 0.5 µg/mL ethidium bromide and visualized on a Doc™ XR+ Gel Documentation System. Bands of interest were cut from the gel and purified using a Zymoclean DNA recovery kit (Zymo Research, Irvine, CA, US) following the manufacturer’s protocols. The purified products were sequenced using a 3500xL genetic analyzer (Applied Biosystems, Foster City, CA, US) in the Synthesis Unit and a Sequencing Unit from the Institute of Biotechnology, UNAM, Mexico.

### Sequence analysis

Sequences were analyzed and manually edited using the Bioedit v.7.0.9 software and the Mega v.7 software^27^. The consensus sequences were compared to other sequences from the GenBank database using the Basic Local Alignment Search Tool of the National Library of Medicine. To determine a hit, the flaviviruses-positive PCR sequences were assessed based on specific criteria, including a total coverage of over 90%, an identity of over 97%, and a minimum length of 223 base pairs. The sequences generated in the present study were deposited in GenBank.

### Phylogenetic analysis

The phylogenetic analysis was performed using the molecular evolutionary genetics analysis (MEGA) v.11 software^28^. The NS5 sequences of viruses representing the *Flavivirus* genus were used. The selection of ZIKV from the American, Asian, and African lineages was obtained from Cabral *et al*.^29^ and Shen *et al*.^30^. The sequences of the cISF, dISF, and NKV groups were obtained from the works of Huang *et al*.^31^ and Rizzo *et al*.^32^. The tree was complemented with the PAFV sequences from Saudi Arabia (MN294940, MN294941, and MN294942) obtained from the National Center for Biotechnology Information (NCBI). In addition, all samples from this study were included. Sequences were aligned using the Clustal W v.2.10 software (EMBL-EBI, Hinxton, Cambridgeshire, UK). Multiple alignment under default parameters and end fragments that were not shared by most of the analyzed sequences were removed.

The phylogenetic reconstruction in MEGA 5 was performed by employing a statistical method based on the Maximum Likelihood method^33^. After analyzing the best tree model, the Kimura 2 (K2) + G + I model was selected. The phylogeny test was performed with the Bootstrap method with 1,000 replications.

### The minimum infection rate

The minimum infection rate (MIR) was estimated as: (number of positive pools/total specimens tested) × 1,000. The MIR is a parameter used to estimate the probability of finding a viral infection in mosquitoes.

## RESULTS

### Adult mosquito collections

A total of 4,321 mosquitoes of five species were captured in 164 households across 17 municipalities in Yucatan State, Mexico (Table 1). Of these, 88 (53.66%) were mosquito-infested. The most common mosquitoes were *Cx. quinquefasciatus* (n = 3,563) and *Ae. aegypti* (n = 734) (Table 1). The adult population of *Cx. quinquefasciatus* consisted of 1,338 females and 2,225 males. *Aedes aegypti* adults were 35.83% female (263 out of 734) and 64.17% male (471 out of 734). The least abundant were *Aedes albopictus* (n = 18), *Aedes taeniorhynchus* (n =5), and *Culex iolambdis* (n =1) (Table 1).

**Table 1:** Households visited and the abundance of female mosquitoes collected by municipality, Yucatan State, Mexico.

Collection site	Nº positive households/total	*Cx. quinquefasciatus*	*Ae. aegypti*	*Ae. albopictus*	*Ae. taeniorhynchu*s	*Cx. iolambdi*s
Merida municipality	14/14	68	96	1	1	0
Celestun municipality	22/22	239	13	0	4	1
Ekmul municipality	12/18	237	78	1	0	0
Tetiz municipality	4/8	138	12	0	0	0
Kinchil municipality	2/8	90	0	0	0	0
Valladolid municipality	1/7	6	0	12	0	0
Chemax municipality	2/8	16	0	0	0	0
Tinum municipality	1/8	5	1	0	0	0
Uman municipality	4/8	26	4	0	0	0
Tahmek municipality	3/8	56	1	0	0	0
Homun municipality	6/8	89	17	0	0	0
Hocaba municipality	2/8	170	4	0	0	0
Cuzama municipality	3/7	24	1	0	0	0
Sotuta municipality	1/7	74	33	1	0	0
Yaxcaba municipality	5/8	88	0	1	0	0
Tizimin municipality	3/10	1	0	0	0	0
Calotmul municipality	3/7	11	3	0	0	0
Total	88/164	1,338	263	16	5	1

### Detection of *Flavivirus* in mosquitoes

In total, 600 female mosquitoes were divided into 60 pools, which corresponded to *Ae. aegypti* (n = 26 pools), *Cx. quinquefasciatus* (n = 33 pools), and *Ae. albopictus* (1 pool). A total of 24 pools of *Flavivirus*-positive PCR were detected in *Ae. aegypti* and subsequently subjected to sequencing using the Sanger method. In total, 12 sequences matched the established quality criteria for assembly (forward and reverse) and were subsequently employed for sequence similarity searches. *Culex quinquefasciatus* and *Ae. albopictus* did not exhibit any positive *Flavivirus* results.

A positive pool of *Ae. aegypti* for the Zika virus (GenBank ID: OR701943) was found in a residence in Merida municipality, Yucatan State, Mexico. The Plebotomus-associated flavivirus was found in *Ae. aegypti* from Celestun municipality (GenBank ID: OR636207), Ekmul municipality (GenBank IDs: OR636209 - OR636212), Homun municipality (GenBank ID: OR636214), Merida municipality (GenBank IDs: OR636204 - OR636206, and OR636208), and Sotuta municipality (GenBank ID: OR636213), of Yucatan State, Mexico (Figure 1).

### Phylogenetic tree

**Figure 2:**
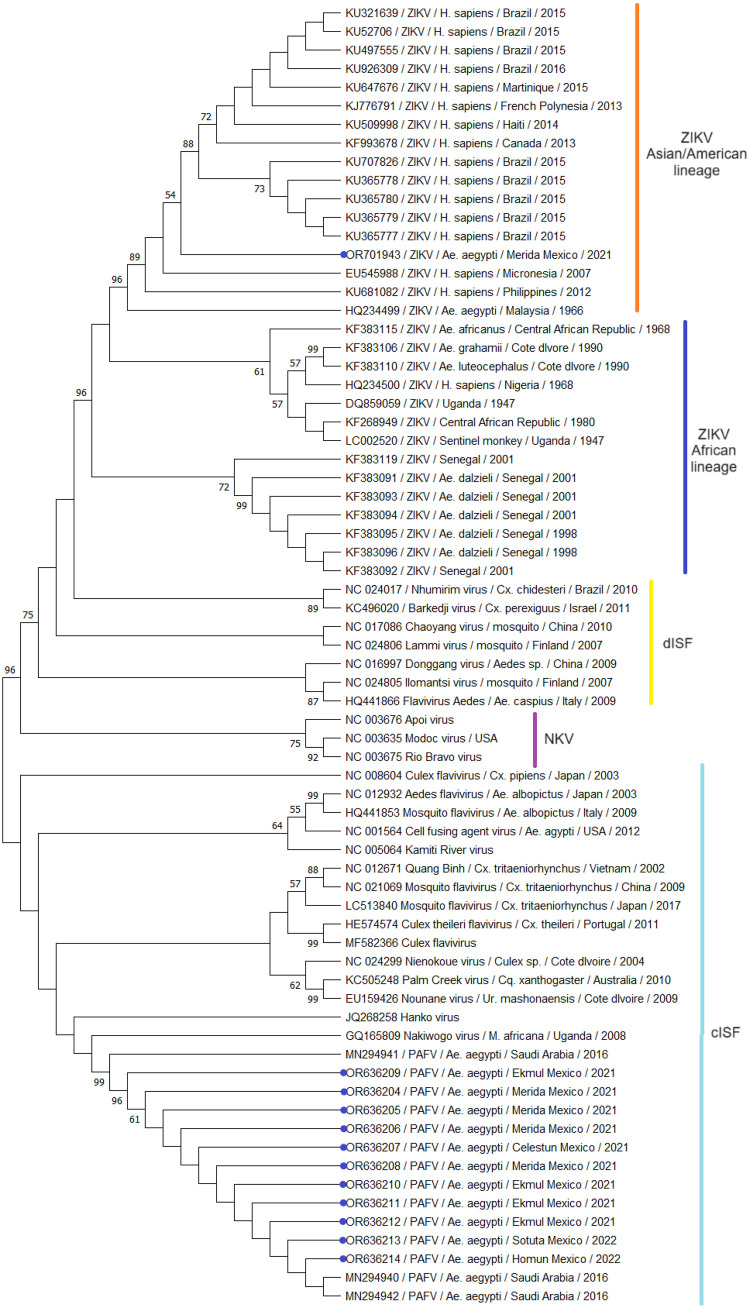
Maximum-likelihood phylogenetic tree of 70 flaviviruses. The tree is based on 219-nt partial NS5 nucleotide sequences. The ML method used the Kimura 2 (K2) + G + I model to generate the tree. Nodes’ numbers indicate bootstrap support values. Labels include the GenBank accession number, corresponding virus name, host, collection site, and date. Sequences identified in this study are marked with blue circles. Zika virus (ZIKV) lineage, classical Insect-specific flaviviruses (cISFs), dual-host-affiliated Insect-specific flaviviruses (dISFs), and viruses with no known vector (NKV).

In total, one out of the 12 *Flaviviridae*-positive pools in *Ae. aegypti* showed an NS5 sequence that was 96.74% the same as the ZIKV strain from Mexico (MT507050.1). In the phylogenetic tree, this sequence was clustered within the Asian lineage’s ZIKV (OR701943) (Figure 2). The following 11 sequences showed nucleotide identity (97.07–99.5%) with a Phlebotomus-associated flavivirus (MN294942.1) from Saudi Arabia (2020). In the phylogenetic tree, these sequences were clustered within classical ISFs (Figure 2).

The minimal infection rate (MIR) for *Flavivirus* was determined to be 40, whereas the Zika virus displayed a MIR of 1.66 and the PAFV displayed a MIR of 18.33.

## DISCUSSION

During the COVID-19 pandemic in Mexico, a significant reduction in the incidence of vector-borne diseases (VBD) was noted. The increased demand for resources dedicated to monitoring and diagnosing COVID-19 may have had an impact on the underreporting of VBD cases^21^. The dengue virus was not identified in our study. However, we found the ZIKV in the mosquito *Ae. aegypti* from November 2021 to May 2022. The finding suggests that the ZIKV was in silent circulation during this period. It is important to acknowledge that this arbovirus exhibited periods of heightened transmission from 2015 to 2017. Currently, instances linked to this particular virus are sporadically documented^13,21^. The estimated MIR for ZIKV was 1.66. The value is similar to that reported for the ZIKV identified in *Ae. aegypti* captured in cemeteries in Merida municipality, Yucatan State, Mexico^24^. However, the value is low compared to the MIR of the dengue virus from *Ae. aegypti* captured from households and schools in the same location^23,25^.

In Mexico, ZIKV RNA has been identified in humans, pigs, and the mosquitoes *Ae. aegypti, Ae. albopictus, Aedes epactius, Ae. taeniorhynchus, Cx. coronator, Cx. erraticus, Cx. lactator, Cx. nigripalpus, Cx. quinquefasciatus, Cx. tarsalis, Cx. thriambus, Culiseta inornata*, and *Cs. particeps* ^24,34-36^.

In Mexico, the dengue virus holds significant importance as a flavivirus, mostly due to its impact on human morbidity and mortality. Moreover, since the reemergence of dengue in Mexico in 1978, there has been a continuous occurrence of outbreaks associated with the four serotypes (DENV 1–4)^13^. Nevertheless, a circulation of flaviviruses in hosts different from humans has been reported within the country. Serological and molecular evidence has been documented, indicating the presence of the West Nile virus and the St. Louis encephalitis virus in birds^16^. The Apoi virus and Modoc virus, which are flaviviruses that specifically infect rodents and present no known arthropod vectors, have been detected in peridomestic rodents within the urban area of Merida municipality, Yucatan State, Mexico^37^.

In this study, we identified a Phlebotomus-associated flavivirus that was distributed across five municipalities in Yucatan State. The Phlebotomus-associated flavivirus was found for the first time in Algeria in a sample of *Phlebotomus perniciosus* ^38^, from which it derives its name. It is the sole ISF that was not named after a mosquito genus. In subsequent investigations, the virus was discovered in *Ae. aegypti* from Saudi Arabia^39^. The importance of ISF in nature has not yet been elucidated; however, it has been suggested that, in situations of co-infection, they may suppress the viral replication of the ZIKV virus and dengue virus^20^. However, evidence also points towards positive interactions between WNV and the ISF Culex flavivirus^18^. An important limitation is that no study has been able to isolate the virus, so its full genome is unknown. However, the evidence of Phlebotomus-associated flavivirus in *Ae. aegypti* of Yucatan State provides an opportunity for future studies to perform the isolation and sequencing to better understand its function.

In the current investigation, flaviviruses were not detected in *Cx. quinquefasciatus* and *Ae. albopictus*. In *Cx. quinquefasciatus* from Yucatan State, researchers have found the Zika virus^35,36^ as well as viruses that are specific to mosquitoes, namely the Culex flavivirus, the T’Ho virus, and the Houston virus^10,15,17^. For *Ae. albopictus* from Yucatan State, there are no reports of flavivirus, and the only ISF report is of AEFV from Japan in 2009^40^.

## CONCLUSION

In conclusion, we found 24 pools of PCR-positive *Flavivirus*, and 12 sequences were assembled during entomo-virological surveillance of *Flavivirus*. The ZIKV was detected in a mosquito pool in an urban area, suggesting that the virus may be silently circulating in Merida municipality, Yucatan State, Mexico. Most of the flavivirus-positive samples were identified as Phlebotomus-associated flavivirus, which shows a wide distribution in Yucatan State and may belong to the virome of *Ae. aegypti* mosquitoes. Future studies should isolate the PAFV to obtain the complete genome and study the function of the species in a mosquito cell line. Therefore, it could be used in competitive superinfection experiments to determine whether PAFV increases or controls the spread of medically important viruses, such as DENV, which is endemic in Mexico.
